# Sodium–Glucose Cotransporter 2 Inhibitors: Does One Size Fit All?

**DOI:** 10.1002/clc.70299

**Published:** 2026-04-11

**Authors:** Paula Cristina Morariu, Maria Mihaela Godun, Marius Traian Dragos Marcu, Mariana Floria

**Affiliations:** ^1^ Department of Internal Medicine Faculty of Medicine University of Medicine and Pharmacy “Grigore T. Popa” Iasi Romania; ^2^ Emergency Clinical Hospital “Saint Spiridon Iasi Romania; ^3^ Clinical Hospital of Pneumophthisiology Iași Romania

## Conflicts of Interest

The authors declare no conflicts of interest.

To the Editor,

I read with great interest the article by Balata et al. [1] about the impact of Empagliflozin versus Dapagliflozin on left ventricular remodeling in heart failure patients. This new standard of treatment in heart failure, sodium–glucose cotransporter 2 inhibitors (SGLT2i), appeared ten years ago. Since 2015 we know that, in patients with type 2 diabetes at high cardiovascular risk, adding Empagliflozin to standard care can decreases significantly the rate of major cardiovascular events and all‐cause mortality compared with placebo [2]. Since 2021 it is well known that SGLT2i reduced significantly the risk of cardiovascular death and heart failure‐related hospitalizations in patients with chronic heart failure and reduced left ventricular ejection fraction [3]. Later, data appeared for preserved left ventricular ejection fraction representing a turning point in current therapy of these patients [3].

In the study of Balata et al. [1], the authors concluded that Empagliflozin, compared with Dapagliflozin, demonstrated more important effects on left ventricular remodeling assessed by standard echocardiographic markers. Even there were no differences between the two groups regarding heart failure hospitalizations or all‐cause mortality, rapid left ventricular filling and sphericity index (i.e. ventricular remodeling) were significantly improved in the Empagliflozin group. The authors stated that it is uncertain whether SGLT2i have differential effects on cardiac structure and function.

Firstly, we consider that there are already some preclinical literature data about these effects of Empagliflozin. Therefore, we do not fully agree that the mechanisms underlying the benefits of Empagliflozin remain completely unclear. Empagliflozin improves cardiac diastolic function (relaxation) in female mice in the setting of obesity and diabetes, as well as profibrotic/prohypertrophic proteins, serum, glucocorticoid regulated kinase 1, epithelial sodium channel profibrosis signaling and associated interstitial fibrosis [4]. In addition, eccentric left ventricular hypertrophy was slightly improved by Empagliflozin, assesed by a reduction in cardiomyocyte cross sectional area [4]. Also, Empagliflozin can ameliorate atrial structural and electrical remodeling as well as improve mitochondrial function and mitochondrial biogenesis [5]. Even in patients with acute decompensated heart failure, immediate addition of Empagliflozin to standard therapy improves echocardiographic parameters of left atrial volume in patients following recompensation [6].

Secondly, NT‐proBNP can be a surrogate marker for the degree of left ventricular wall stress. In this study, NT‐proBNP was higher in Empagliflozin group and left ventricular remodeling was significantly greater in this group; not only the remodeling was more important but also it was carried out faster. We think it's not surprising, taking into consideration the preclinical and clinical data [4, 5]. It was stated, already, that the effects of Empagliflozin appear much faster than Dapagliflozin (12 days in EMPEROR Reduced Trial vs 28 days in DAPA‐HF trial), which supports a possible more rapid effect on left ventricular remodeling. The analysing of the time to significant treatment effects in the most major heart failure drug clinical trials demonstrated that empagliflozin need 12 days and Dapagliflozin 28 days [7].

Thirdly, Empagliflozin group of this study published by Balamata M et al [1]. included significantly more patients with obesity and diabetes mellitus. Regarding the methodology, daily dose for Empagliflozin was either 10 or 25 mg, and for Dapagliflozin was a fixed dose of 10 mg daily. It is important taking into consideration that an estimated glomerular filtration cut‐off of 30 mL/min/1.73 m^2^ was chosen [1]. Some patients who were eligible for Empagliflozin were excluded, estimated glomerular filtration cut‐off up to which it was allowed was 20 mL/min/1.73 m^2^. We believe that these parameters in the methodology could constitute factors that can make a difference in obtaining results.

In conclusion, one size does not fit all. There are probably differences in efficacy or mechanisms of action between SGLT2i. Preclinical data support these results.

## Data Availability

Data sharing not applicable – no new data were generated in this manuscript.

## References

[1] M. Balata , M. U. Becher , M. Hassan , et al., “Impact of Empagliflozin Versus Dapagliflozin on Left Ventricular Remodeling in Heart Failure Patients: A 1‐Year Comparative Study,” Clinical Cardiology 48, no. 9 (2025): e70192, 10.1002/clc.70192. 40966463 PMC12445616

[2] B. Zinman , C. Wanner , J. M. Lachin , et al., “Empagliflozin, Cardiovascular Outcomes, and Mortality in Type 2 Diabetes,” New England Journal of Medicine 373, no. 22 (2015): 2117–2128, 10.1056/NEJMoa1504720. 26378978

[3] M. Authors/Task Force , T. A. Mcdonagh , M. Metra , et al., “2021 ESC Guidelines for the Diagnosis and Treatment of Acute and Chronic Heart Failure: Developed by the Task Force for the Diagnosis and Treatment of Acute and Chronic Heart Failure of the European Society of Cardiology (ESC). With the Special Contribution of the Heart Failure Association (HFA) of the ESC,” European Journal of Heart Failure 24, no. 1 (2022): 4–131, 10.1002/ejhf.2333. 35083827

[4] J. Habibi , A. R. Aroor , J. R. Sowers , et al., “Sodium Glucose Transporter 2 (SGLT2) Inhibition With Empagliflozin Improves Cardiac Diastolic Function in a Female Rodent Model of Diabetes,” Cardiovascular Diabetology 16, no. 1 (2017): 9, Published 2017 Jan 13. 10.1186/s12933-016-0489-z. 28086951 PMC5237274

[5] J. Bogoviku , T. D. Nguyen , J. G. Westphal , et al., “Acute Effects of Empagliflozin on Left Atrial and Ventricular Filling Parameters Using Echocardiography‐A Subanalysis of the EMPAG‐HF Trial,” European Heart Journal—Cardiovascular Pharmacotherapy 11, no. 2 (2025): 190–197, 10.1093/ehjcvp/pvaf003. 40037298 PMC11905748

[6] Q. Shao , L. Meng , S. Lee , et al., “Empagliflozin, a Sodium Glucose co‐transporter‐2 Inhibitor, Alleviates Atrial Remodeling and Improves Mitochondrial Function in High‐Fat Diet/Streptozotocin‐Induced Diabetic Rats,” Cardiovascular Diabetology 18, no. 1 (2019): 165, Published 2019 Nov 28. 10.1186/s12933-019-0964-4. 31779619 PMC6882319

[7] D. Bismpos , J. Wintrich , J. Hövelmann , and M. Böhm , “Latest Pharmaceutical Approaches Across the Spectrum of Heart Failure,” Heart Failure Reviews 29, no. 3 (2024): 675–687, 10.1007/s10741-024-10389-8. 38349462 PMC11035443

